# Human placenta-derived endothelial progenitor cells: an animal-free culture system for efficient expansion

**DOI:** 10.1186/s40659-025-00625-2

**Published:** 2025-07-02

**Authors:** Shengnan Yuan, Mengrou Li, Junhao Wang, Wen Ju, Yujin Huang, Yue Li, Haohan Fan, Lingyu Zeng

**Affiliations:** 1 Key Laboratory of Bone Marrow Stem Cell, Xuzhou Medical University, Xuzhou, Jiangsu China; 2 Blood Diseases Institute, Xuzhou Medical University, Xuzhou, Jiangsu China; 3 School of Medical Technology, Xuzhou Medical University, Xuzhou, Jiangsu China; 4 Department of Hematology, The Affiliated Hospital of Xuzhou Medical University, Xuzhou, Jiangsu China

**Keywords:** EPCs, Placenta, Animal-free culture system, Wound repair

## Abstract

**Background:**

Endothelial progenitor cells (EPCs) play a critical role in vasculogenesis and vascular repair, but their clinical application is hindered by challenges such as cell purity, quantity, and reliance on fetal bovine serum (FBS). This study developed an animal-free system for isolating, induction, and expanding EPCs from the human placenta, evaluating their potential for wound repair.

**Methods:**

Mononuclear cells (MNCs) were isolated from full-term placenta and induced into EPCs using an animal-free medium supplemented with bFGF, IGF, and VEGF. EPCs were characterized by flow cytometry for markers CD133, CD34, and VEGFR2, while CD31 and CD45 served as negative markers. Functional assays, including Ac-LDL uptake, migration, and tube formation, confirmed EPC properties. The wound-repair potential was assessed in a mouse model.

**Results:**

The induced EPCs exhibited high purity (> 95%) and expressed CD133, CD34, and VEGFR2 while being negative for CD31 and CD45. The system yielded 1 × 10⁸ EPCs from 10 g of placental tissue, demonstrating high proliferative capacity. Functional assays confirmed robust tube formation, migration, and Ac-LDL uptake in vitro. In vivo, EPCs significantly enhanced wound repair.

**Conclusions:**

In conclusion, human placenta-derived EPCs cultured in an animal-free system displayed high purity, self-renewal capacity, and functional efficacy, making them a promising cell source for therapeutic applications, particularly in wound repair.

**Supplementary Information:**

The online version contains supplementary material available at 10.1186/s40659-025-00625-2.

## Background

Endothelial progenitor cells (EPCs) are the precursor cells of vascular endothelial cells and possess the potential to differentiate into mature endothelial cells [1]. EPCs play a pivotal role in vascular repair, regeneration, and the maintenance of vascular homeostasis, making them a focal point of research in regenerative medicine. Numerous studies have demonstrated the safety and efficacy of EPC-based therapies, particularly in the treatment of critical limb ischemia and cardiovascular diseases [2, 3]. However, the clinical application of EPCs faces significant challenges, including the high cell doses required for therapeutic efficacy and the low frequency of EPCs in available sources, such as umbilical cord blood, adult peripheral blood (PB), and bone marrow (BM) [4–6]. These limitations hinder the widespread adoption of EPC-based therapies in clinical settings, necessitating the development of efficient methods for in vitro expansion of EPCs.

A further complication arises from the widespread use of fetal bovine serum (FBS) in current EPC culture protocols [7]. While FBS is a common supplement for culturing primary cells and cell lines, it carries risks of microbiological contamination, including viruses, prions, bacteria, mycoplasma, yeast, fungi, and endotoxins [8, 9]. These safety concerns, coupled with ethical issues, underscore the need for animal-free culture systems to produce clinically viable EPCs.

To address these challenges, we explored human placental tissue as a novel and abundant source of EPCs. The mononuclear cells (MNCs) from various tissues, including PB, BM, umbilical cord, and placental tissue, has been well-established [10–14]. The placenta is a highly vascularized organ rich in progenitor cells, including EPCs, and has been increasingly recognized as a promising alternative to traditional sources such as PB or BM [14, 15]. MNCs can be induced into functional cells, such as mesenchymal stem cells (MSCs), macrophages, and induced pluripotent stem cells (iPSCs) with special ingredient additions in the culture system [16, 17]. MNCs derived from human umbilical cord blood and BM have been induced into EPCs with the use of specialized growth medium and fetal bovine serum [10, 12]. The isolation of MNCs via density gradient centrifugation is a well-established method for enriching progenitor cell populations while eliminating erythrocytes and granulocytes [18].

For EPC induction and expansion, we developed an animal-free culture system supplemented with key growth factors—basic fibroblast growth factor (bFGF), insulin-like growth factor (IGF), and vascular endothelial growth factor (VEGF)—selected based on their well-documented roles in vascular endothelial differentiation and progenitor cell maintenance [10, 12]. The identity of EPCs was confirmed through established markers, including CD133, CD34, and vascular endothelial growth factor receptor 2 (VEGFR2), which are widely accepted for defining progenitor cells with endothelial potential [10, 19]. CD133 is a well-characterized marker of primitive stem/progenitor cells, including hematopoietic and endothelial progenitors. CD133 is expressed on immature EPCs but lost during differentiation into mature endothelial cells [20]. CD34 is a classical marker of hematopoietic and endothelial progenitor cells. It is retained in early EPCs but downregulated in differentiated endothelial cells [21]. VEGFR2 is the primary receptor for VEGF and is specific to endothelial lineage and absent in hematopoietic stem cells [22]. CD31 (also named PECAM-1) is expressed on mature endothelial cells but is typically absent or weakly expressed in early EPCs, which are progenitor cells with distinct phenotypic and functional properties compared to mature endothelial cells. CD31 and CD45 were used as negative markers to exclude mature endothelial cells and hematopoietic lineages, respectively [18, 23]. Positive CD133/CD34/VEGFR2 and negative CD31/CD45 confirm the endothelial progenitor identity. Functional characterization, including acetylated low-density lipoprotein (Ac-LDL) uptake, migration, and tube formation assays, was performed in line with consensus standards for validating EPC functionality [18].

This study aims to establish a reliable method for culturing EPCs from human placental tissue in an animal-free system, addressing the limitations associated with high cell doses and FBS use. By characterizing EPCs using specific markers and growth factors, we ensured their endothelial lineage commitment after multiple passages. Our findings demonstrated that this animal-free culture system is a promising and effective approach for the clinical application of EPCs, offering a safer and more ethical alternative to traditional methods.

## Methods

### Management of placenta

Full-term human placental tissue (10 g) was minced into small pieces. The tissue fragments were enzymatically digested using a solution containing 10 mg of Type II collagenase (derived from Clostridium histolyticum; 40508ES76, Yeasen Biotechnology, Shanghai, China), 10 mL of EBM-2 basal medium (CC-3156, LONZA, Basel, Switzerland), and 10 mL of recombinant trypsin (obtained from microbial fermentation; A1285901, Thermo Fisher, Massachusetts, USA). The digestion was performed in a 37 °C shaker for 1.5 h. Both collagenase and trypsin used in this study were of non-animal origin. After digestion, the cell suspension was filtered through sterile gauze to remove undigested tissue fragments. The filtrate was then diluted with 20 mL of phosphate-buffered saline (PBS) and centrifuged at 1500 rpm for 5 min. The supernatant was discarded, and the cell pellet was washed twice with PBS under the same centrifugation conditions.

### Isolation of mononuclear cells (MNCs)

Lymphocyte separation solution (GE Healthcare, Milwaukee, WI, USA) and cell suspension above were added to the centrifuge tube. The cell suspension was gently added to the upper layer along the tube wall to avoid mixing (the interface between the two fluids was clear). The tubes were centrifuged at 1500 rpm for 30 min at room temperature. The liquid was layered from top to bottom into a PBS layer, white membrane layer (MNCs), separated liquid layer, erythrocyte and granulocyte layer. The white membrane layer was carefully drawn to a new centrifuge tube with PBS and centrifuged at 1500 rpm for 5 min. Finally, 1.0 × 10⁷ MNCs were obtained for subsequent experiments.

### Cell induction and culture in an animal-free system

All reagents used in this study were of non-animal origin. The basal medium for cell culture was EBM-2, supplemented with 5% platelet lysate (S300-100, BDBIO, Zhejiang, China). Additionally, the following growth factors and supplements were added to the culture system: human VEGF (kx-GMP-043), IGF (kx-GMP-034), bFGF (kx-GMP-007) (all purchased from Beijing Kexin Biology, Beijing, China), hydrocortisone (HY-N0583, MCE, New Jersey, USA), and ascorbic acid (HY-B0166, MCE, New Jersey, USA). The 1.0 × 10⁷ MNCs isolated as described above were seeded into fibronectin-coated culture dishes. After 24 h, the culture medium was replaced with a fresh medium, and then growth factors were replenished every 48 h during the culture period. Cell expansion was initiated when the cell density reached approximately 85% confluence.

### Flow cytometry analysis

Single-cell suspensions were prepared and stained with fluorochrome-conjugated antibodies for 1 h in ice-cold PBS. The following antibodies were used: anti-human CD133, anti-human CD34, anti-human VEGFR2, anti-human CD31, and anti-human CD45 (all purchased from BioLegend, California, USA). After staining, cells were analyzed using the LSR Fortessa™ cell analyzer (BD Biosciences, New Jersey, USA). Data were processed and analyzed using FlowJo software, version 10.1 (Tree Star).

### Acetylated low-density lipoprotein (Ac-LDL) uptake assay

For the Ac-LDL uptake assay, 20,000 EPCs were seeded per dish and cultured for 20 h. After removing the culture medium, cells were washed once with PBS, and 1 mL of fresh medium was added to each dish. Subsequently, 50 µg of red fluorescent DiI-Ac-LDL (MP6013, MKbio, Shanghai, China) was added to each dish, and the cells were incubated at 37 °C for 4 h. Following incubation, the cells were washed once with PBS to remove unbound DiI-Ac-LDL. For cell fixation, 1 mL of 2% paraformaldehyde was added to each dish and incubated at room temperature for 10 min. After fixation, the cells were washed once with PBS. To label the cells, 1 mL of PBS and 2 µL of FITC-conjugated Ulex europaeus agglutinin 1 (FITC-UEA-1; MP6308, MKbio, Shanghai, China) were added to each dish and incubated at room temperature for 1 h. FITC-UEA-1 binds specifically to α-L-fucose residues on endothelial cell surface glycoproteins, serving as a complementary marker to DiI-Ac-LDL uptake for confirming endothelial lineage. Dual-stained cells positive for both DiI-Ac-LDL and FITC-UEA-1 were identified as EPCs [18]. The cells were then washed once with PBS to remove excess FITC-UEA-I. Finally, 1 mL of PBS, 1 µL of Hoechst 33,342 (5 mg/mL; HY-15559, MCE, New Jersey, USA) was added to each dish for nuclear staining. After incubating at room temperature for 10 min, the cells were washed once with PBS. Images were acquired using a confocal microscope (ZEISS LSM 880, Oberkochen, Germany).

### Cell migration assay

For the cell migration assay, EPCs were seeded into a 6-well plate and cultured until the cell density reached 90% confluence. A vertical scratch was made on the cell monolayer using a sterile pipette tip. The cells were then washed twice with pre-warmed PBS to remove detached cells. Subsequently, EBM-2 basal medium was added to the wells, and images of the scratch were captured at the 0-hour time point. The cells were further incubated at 37 °C, and images were taken at regular intervals to monitor cell migration into the scratched area. The migration rate was calculated by measuring the change in the scratch width over time using the Image J (1.8.0) analysis software.

### Tube-formation assay

For the tube-formation assay, the matrix gel (BD, New Jersey, USA) was thawed overnight at 4 °C, and a 96-well plate was pre-chilled at -20 °C overnight. The following day, the 96-well plate was placed on ice, and 100 µL of the thawed matrix gel was added to each well. The plate was then incubated at 4 °C for 20 min to ensure even distribution of the gel, followed by incubation at 37 °C for 30 min to allow the gel to solidify. Next, 10,000 EPCs resuspended in 100 µL of PBS were carefully added to the center of each well. The plate was then transferred to a 37 °C cell culture incubator. Tube formation was observed under a microscope, and images were captured after 5 h of incubation. Five random fields of view per well were analyzed at 10× magnification. Three replicate wells were assessed for each experiment from five different placentas.

### EPCs repair assay

Mice (C57BL/6J, male, 8-week-old, 22.3 ± 1.5 g) were anesthetized via intraperitoneal injection of ketamine/xylazine cocktail (100/10 mg/kg) dissolved in sterile saline. Within 2 to 3 min after the injection, the mice gradually entered anesthesia, showing loss of consciousness, loss of the reversal reflex, and slower breathing and heart rate. Dorsal hair was removed with electric clippers followed by depilatory cream. One full-thickness excisional wound (10 mm diameter) was created per mouse using a sterile biopsy punch, preserving panniculus carnosus. Hemostasis was achieved with sterile gauze compression. EPCs (1 × 10^5^ cells in 200 µL PBS) or vehicle (PBS) were administered via tail vein injection post-wounding for one time. Wound size was analyzed every other day from day 0 to day 10. The wound healing rate was calculated by measuring the wound area using Image J (1.8.0). Mice were individually housed in IVC cages (Tecniplast GM500) under specific pathogen-free (SPF) conditions (22 °C, 55% humidity, 12:12 light cycle) at Xuzhou Medical University. The study was approved by Xuzhou Medical University and conducted in full compliance with ARRIVE 2.0 and the NIH Guide for Care and Use of Laboratory Animals.

### CO₂ euthanasia

Mice were individually placed in the chamber. CO₂ was introduced at a gradual displacement rate of 20% chamber volume per minute. ≥70% CO₂ was maintained for 5 min after respiratory arrest, following AVMA guidelines. Death was confirmed by the absence of a heartbeat and pupillary reflex. All procedures were approved by the Ethics Committee of Xuzhou Medical University.

### Statistical analysis

Statistical analyses were performed using GraphPad Prism 9.0 (GraphPad Software, La Jolla, CA, USA). The normality of data distribution was assessed by the Shapiro-Wilk test, and the homogeneity of variances was confirmed via Levene’s test. For comparisons between two groups, two-tailed unpaired Student’s *t*-tests were applied. Data represent mean ± SD from 5 independent placental donors with 3 technical replicates each. *p*-values were adjusted using Bonferroni correction for multiple comparisons. A probability threshold of *p* < 0.05 was considered statistically significant. *p < 0.05, **p < 0.01, ***p < 0.001, ****p < 0.0001.

## Results

### MNCs were isolated from the placenta and induced in an animal-free culture system

**Fig. 1:**
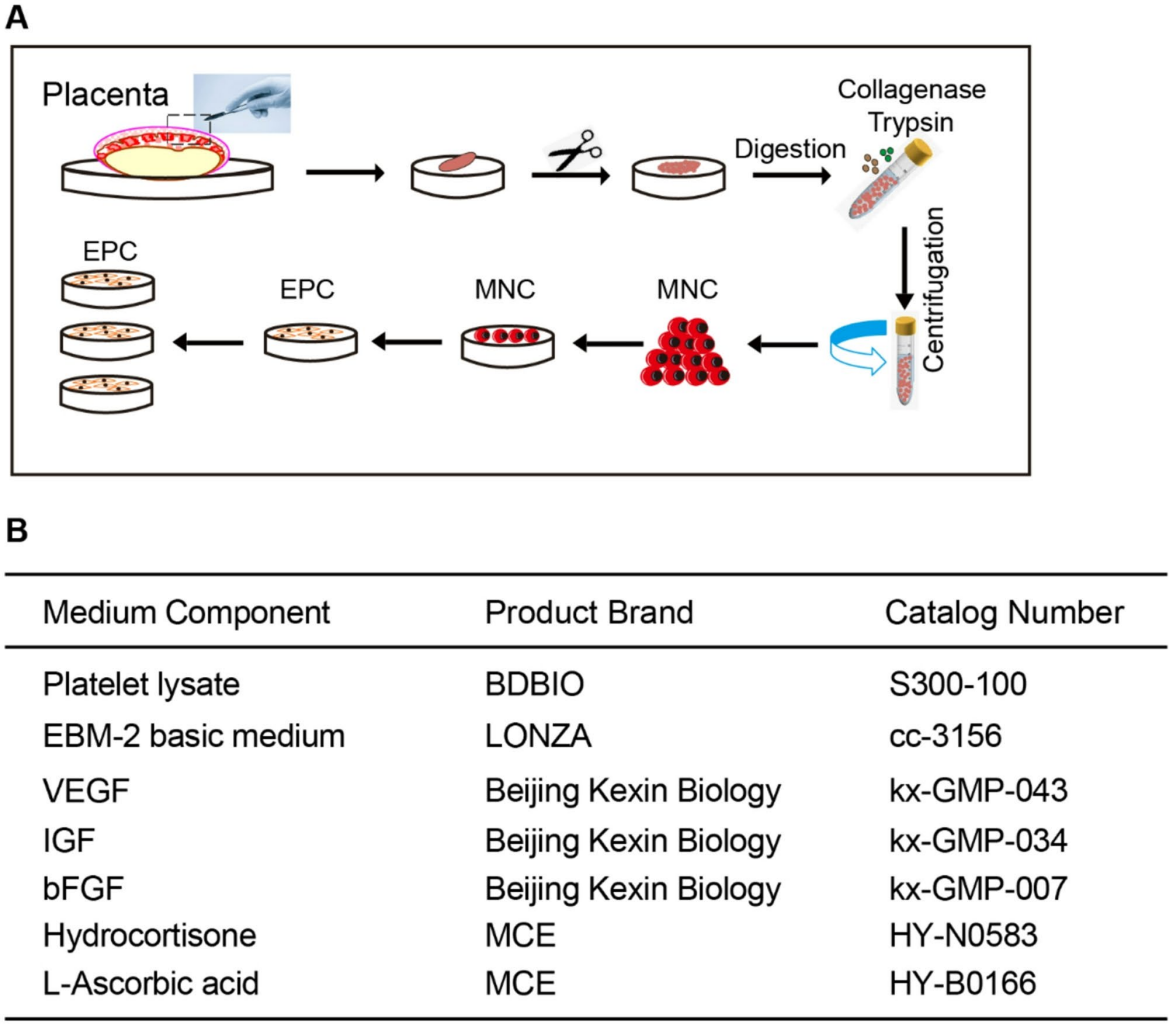
Schematic representation of the EPCs isolation and culture process (**A**) Steps for isolating EPCs from human placental tissue. Mononuclear cells (MNCs) were extracted from the placenta through enzymatic digestion using collagenase and trypsin, followed by centrifugation. (**B**) Composition of the animal-free culture medium used for EPC induction. Key components include platelet lysate, EBM-2 basic medium, and growth factors (VEGF, IGF, bFGF), along with additional supplements such as hydrocortisone and L-ascorbic acid. The specific product brands and catalog numbers are listed for each component

MNCs used in the experiment were isolated from full-term human placenta. A 10 g portion of the placenta was finely minced, and then 10 mg of Type II collagenase, 10 mL of EBM-2 basal medium, and 10 mL of trypsin were added. The mixture was incubated in a shaker at 37 °C for 1.5 h. The cell suspension was obtained after filtration through gauze and then resuspended in 20 mL of PBS. Lymphocyte separation solution and cell suspension were layered in a centrifuge tube, with the cell suspension gently added on top. After centrifugation at 1500 rpm for 30 min, distinct layers formed: PBS, white membrane (mononuclear cells, MNCs), separation solution, and erythrocyte/granulocyte layers. The white membrane layer was carefully collected and resuspended in PBS. The cells were washed twice, yielding 1.0 × 10^7^ MNCs for EPC induction (Fig. 1A). We examined the percentages of CD34/CD45/CD31 in MNCs from five different placentas. The percentages of CD34^+^ cells in MNCs from five different placentas were about 3.75% (Supplemental Fig. 1A-B). Less than 5% contamination with CD45^+^ hematopoietic cells or CD31^+^ mature endothelial cells was examined by flow cytometry (Supplemental Fig. 1C-F). Wright-Giemsa staining of isolated MNCs showed characteristic lymphocyte-like morphology with a high nuclear-to-cytoplasmic ratio. Erythrocytes and granulocytes were rarely observed (Supplemental Fig. 1G). None of the reagents used in the culture medium for EPC induction were of animal origin. The basic medium used was EBM-2, supplemented with 5.0-7.5% platelet lysate. Human recombinant cytokines (including VEGF, IGF, and bFGF), hydrocortisone, and ascorbic acid were added to the culture system (Fig. 1B). The 1.0 × 10^7^ MNCs prepared as described above were seeded into a fibronectin-coated dish. The growth factors were replenished every 48 h, and cell expansion was initiated when the cell density reached 85% confluence.

### EPCs morphological feature could be maintained from P0 to P4 generation

**Fig. 2:**
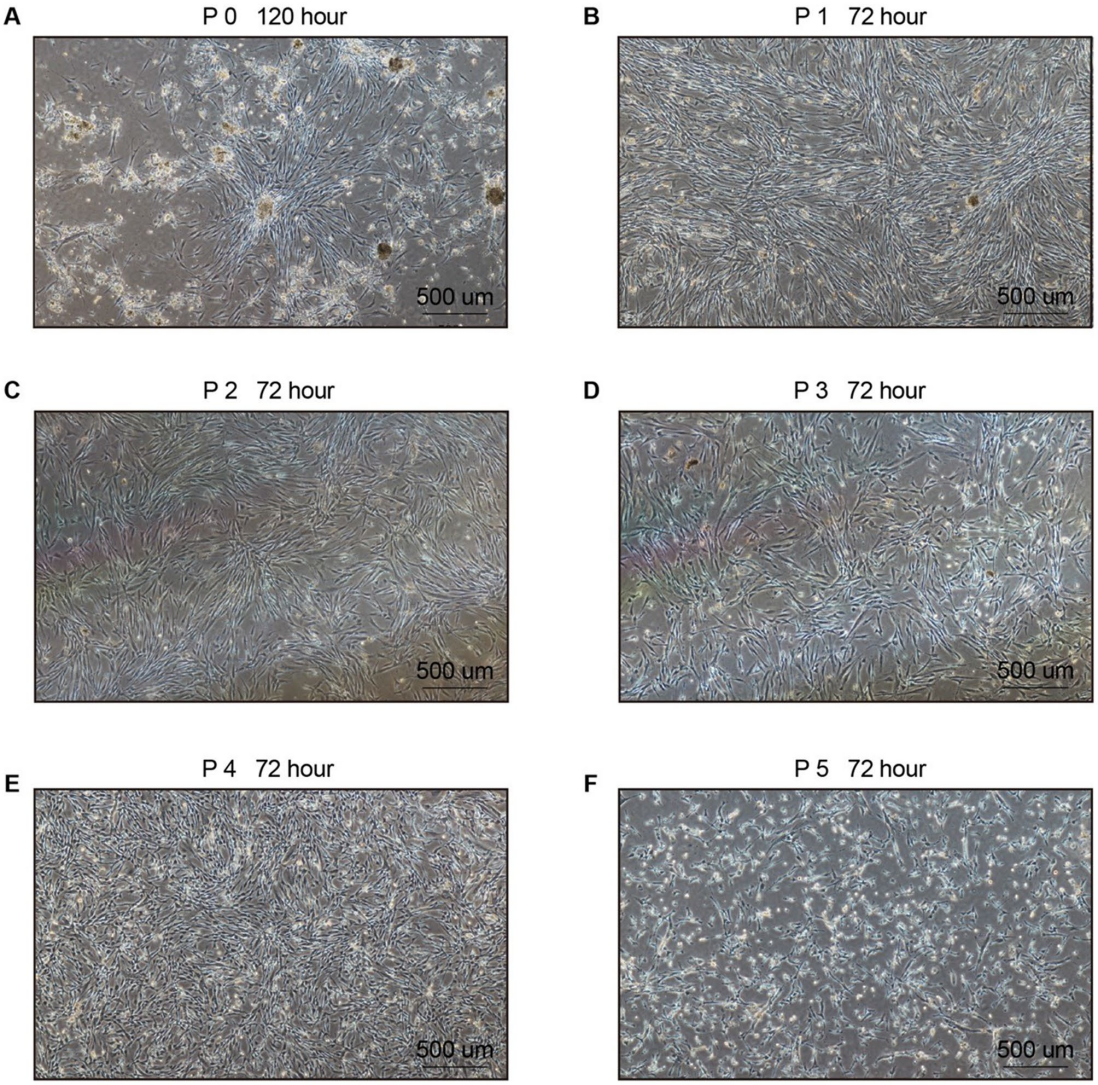
Images of EPCs at different generations from P0 to P5. (**A**) Image at P0 after 120 h. (**B**) Image at P1 after 72 h. (**C**) Image at P2 after 72 h. (**D**) Image at P3 after 72 h. (**E**) Image at P4 after 72 h. (**F**) Image at P5 after 72 h. Scale bars represent 500 μm

P0 EPCs, derived from MNCs isolated from the placenta, still contained a small population of non-EPC cells and exhibited clustered growth (Fig. 2A). P0 EPCs were trypsinized and sub-cultured. EPCs from P1 to P4 displayed a uniform spindle-shaped morphology, with a denser and more organized growth pattern (Fig. 2B-E). These morphological characteristics were retained until the P4 generation; however, P5 cells lost their EPC-like morphology and adopted a mesh-like growth pattern, accompanied by a markedly reduced growth rate (Fig. 2F). These findings suggested that EPCs from different generations exhibit distinct morphological characteristics, with cells from P1 to P4 displaying a more homogeneous EPC phenotype. To further verify the identity of these cells, flow cytometry analysis was performed on cells from P0 to P5.

### The purity of surface markers varied in EPCs from P0 to P5

**Fig. 3:**
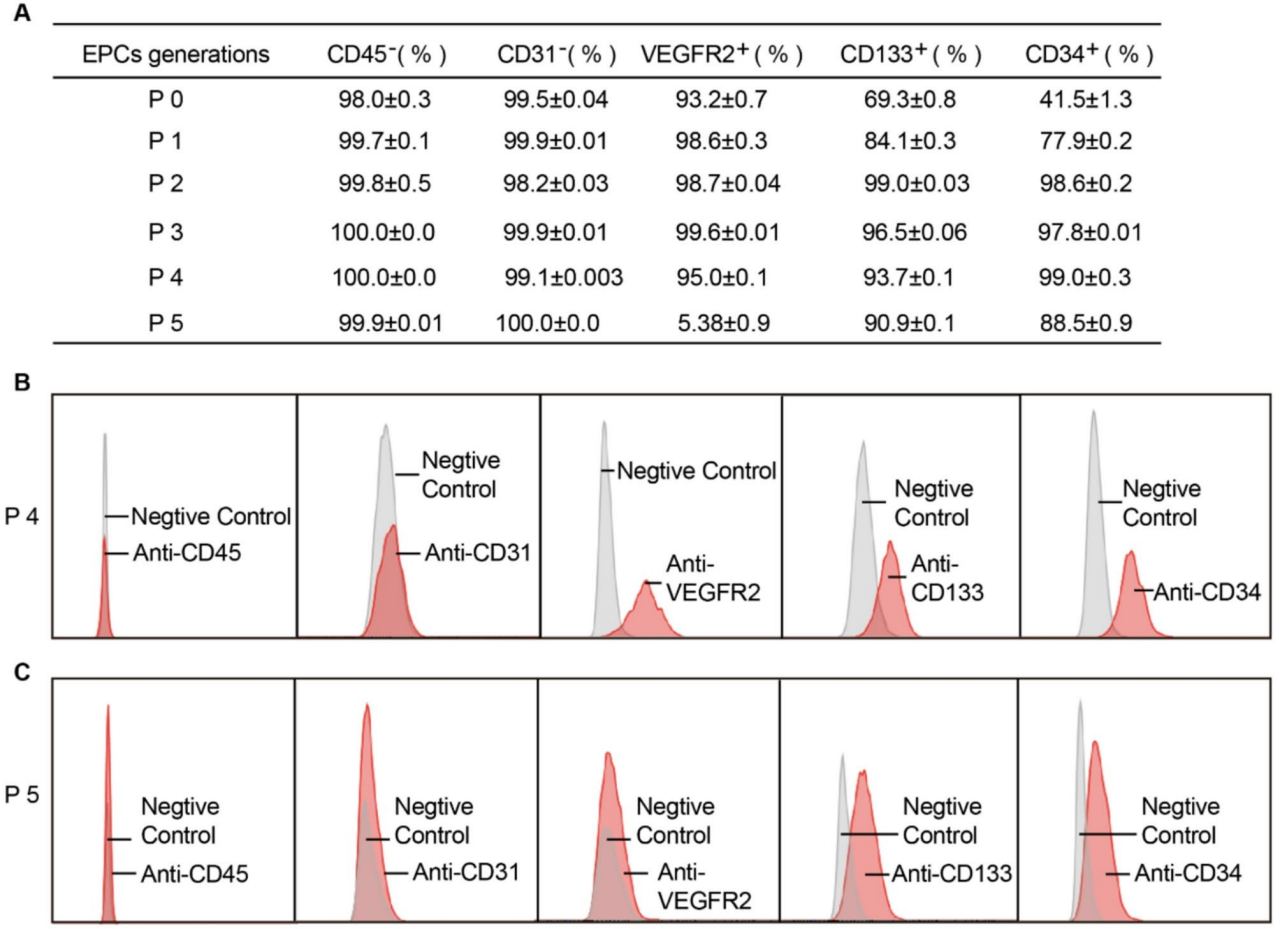
Analysis of marker expression across different culture generations. (**A**) Percentage of cells expressing CD45, CD31, VEGFR2, CD133, and CD34 at various passages (P0 to P5). (**B**) Negative control and specific antibody staining for CD45, CD31, VEGFR2, CD133, and CD34 at P4. (**C**) Negative control and specific antibody staining for CD45, CD31, VEGFR2, CD133, and CD34 at P5. The data demonstrate the expression profile of key markers during the culture process

EPCs were defined as CD133^+^/CD34^+^/VEGFR2^+^ while negative for CD31 (mature endothelial marker) and CD45 (hematopoietic marker), consistent with their progenitor status. The expression levels of surface markers varied across EPCs from P0 to P5. The percentages of CD31 and CD45 negative cells in EPCs from P0 to P5 were all close to 100%. The percentages of CD133 and CD34 in P0 EPCs were 69.3% and 41.5%, respectively, indicating a lower purity of P0 EPCs. The percentages of CD133 (84.1%) and CD34 (77.9%) in P1 EPCs increased compared to the P0 generation. The percentages of VEGFR2 in P0 and P1 EPCs exceeded 93% (Fig. 3A). The percentages of CD133, CD34, and VEGFR2 in P2 to P4 EPCs were all above 93.0%, while the absence of CD31 and CD45 was consistently close to 100% (Fig. 3A). However, the percentage of VEGFR2 in P5 EPCs dropped to 5.38%, despite the percentages of CD133 and CD34 remaining high at 90.0% and 88.5%, respectively (Fig. 3A). Representative flow cytometry plots for P4 and P5 EPCs are also presented (Fig. 3B-C). These results suggest that EPCs from P2 to P4 generations exhibited high purity.

### EPCs cultured in the animal-free system have enough cell output and good function

**Fig. 4:**
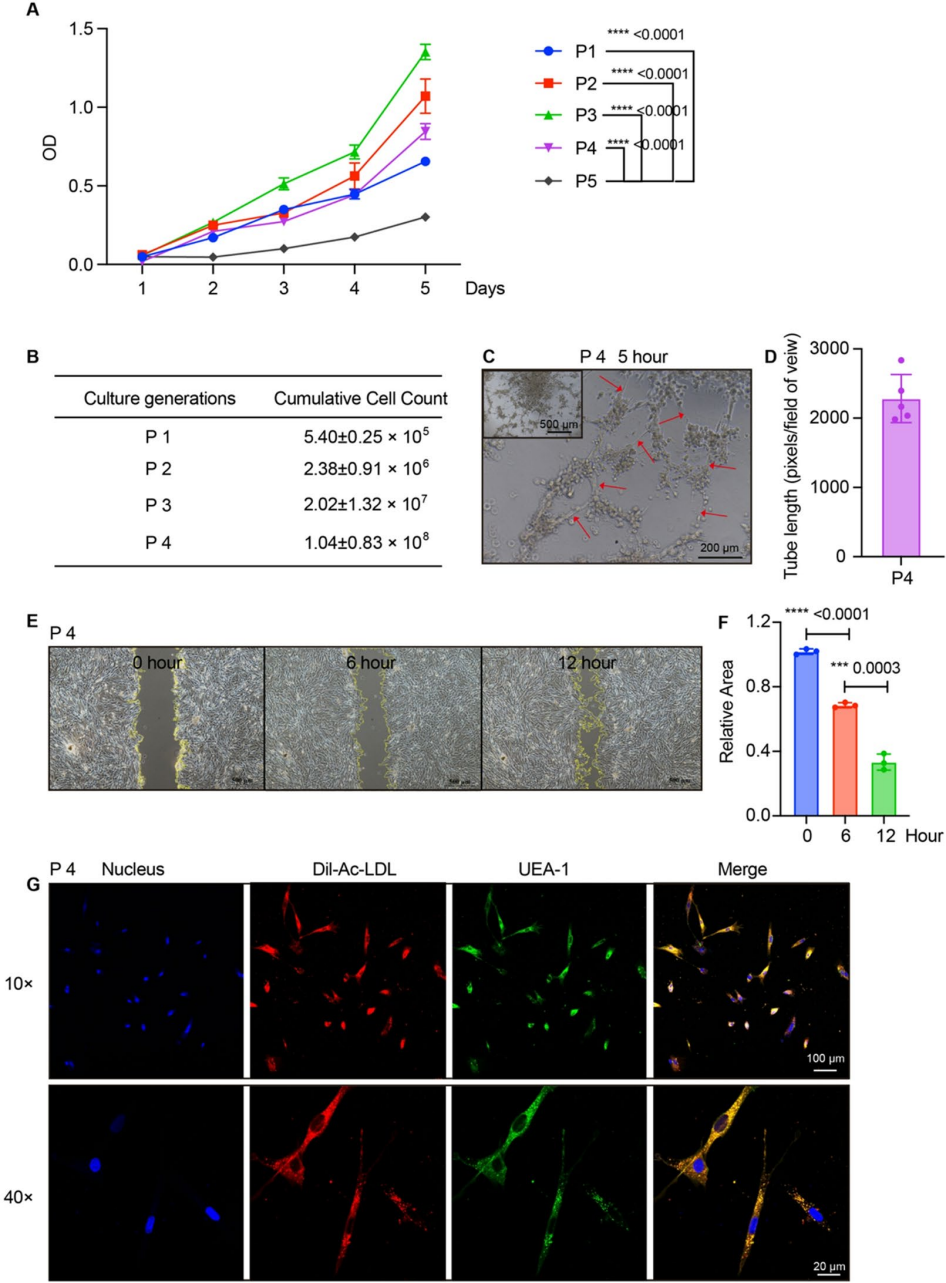
Cell proliferation and functional characterization. (**A**) Cell proliferation examined by CCK-8. *n* = 5. Student’s *t*-test. *p < 0.05, **p < 0.01, ***p < 0.001, ****p < 0.0001. (**B**) Cumulative counts of cell passages from P1 to P4. (**C**-**D**) Representative images of tube formation at P4 after 5 h (**C**) with scale bars indicating 200 μm and quantification of the tube length (**D**). *n* = 5. (**E**-**F**) Unhealed area of cell migration over time (0, 6, and 12 h) with scale bars indicating 500 μm (**E**) and quantitative analysis of unhealed area (**F**). *n* = 3. Student’s *t*-test. *p < 0.05, **p < 0.01, ***p < 0.001, ****p < 0.0001. (**G**) Fluorescence images showing EPC nucleus, Dil-Ac-LDL uptake, UEA-1 binding, and merged image, indicating functional properties of the cells. Scale bars indicate 100 μm (10×) and 20 μm (40×)

To investigate the metabolic changes, glucose uptake and lactate production were analyzed. P3 EPCs demonstrated the highest glucose (2-Deoxy-D-glucose, 2-DG) uptake capacity coupled with the lowest lactate production (Supplemental Fig. 2A-B), indicating efficient oxidative metabolism. This was closely followed by P2 EPCs, which also displayed favorable metabolic profiles. In contrast, P5 EPCs exhibited the lowest glucose uptake and the highest lactate accumulation (Supplemental Fig. 2A-B), suggesting a shift toward glycolytic metabolism—a hallmark of cellular aging or stress. The proliferative capacity of EPCs from P1-P5 was assessed using the CCK-8 assay for 5 days (Fig. 4A). All passages exhibited an increase in cell proliferation. Notably, P3 EPCs demonstrated robust growth kinetics, while P5 showed slow proliferation. The cumulative count of cells from the P1 to P4 generations has finally reached 1.04 × 10^8^, while the initial MNCs were obtained from only 10 g placental tissue (Fig. 4B). The functional capabilities of EPCs, including tube formation, cell migration, and Ac-LDL uptake, were evaluated. Tube formation (for example, P4 EPCs) was evident after 5 h of culture (Fig. 4C-D). Cell migration (P4 EPCs) was observed after 6 h, with increased migration activity evident after 12 h (Fig. 4E-F). The Ac-LDL uptake capability of EPCs was confirmed through immunofluorescence staining (Fig. 4G). These results indicate that EPCs cultured in the animal-free system demonstrated robust cell expansion and functional properties.

### The function of EPCs varied in different generations

**Fig. 5:**
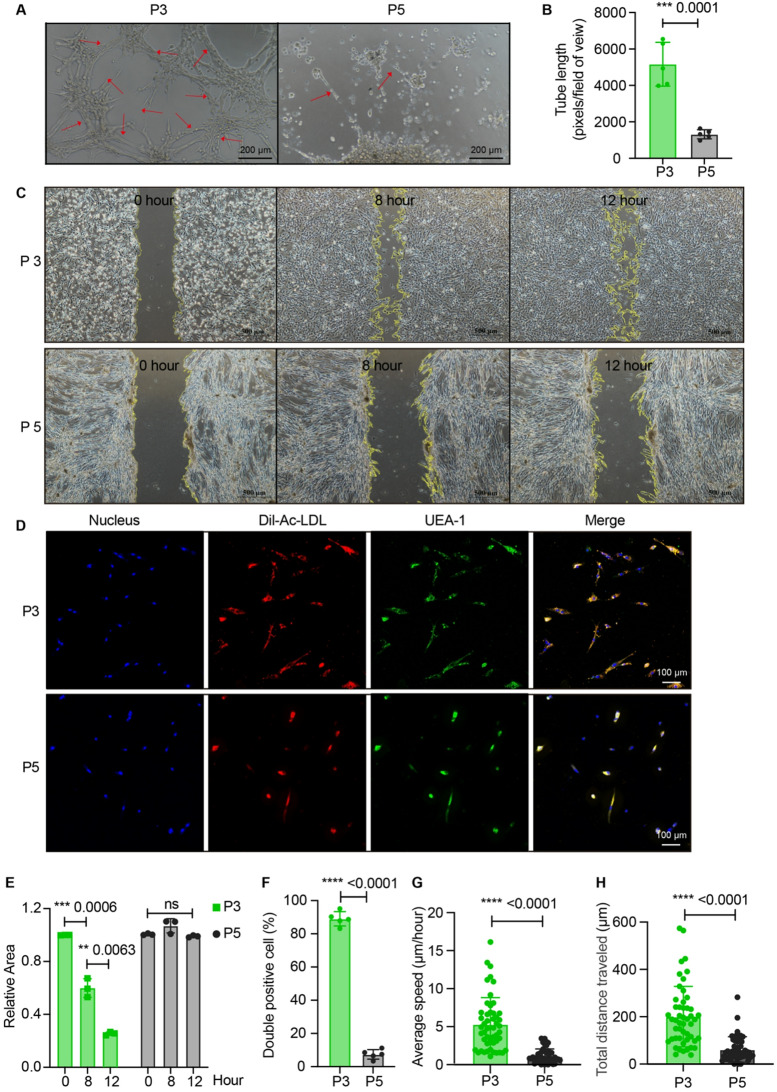
The function of EPCs varied in different generations, with P5 showing reduced EPCs properties. (**A**-**B**) Tube formation assay: Representative images (**A**) of tube formation (pixels of tubes per field of view) and quantification of the tube length (**B**) of P3 EPCs and P5 EPCs after 5 h, with scale bars indicating 200 μm. *n* = 5. Student’s *t*-test. *p < 0.05, **p < 0.01, ***p < 0.001, ****p < 0.0001. (**C**) Cell migration assay: Representative images of P3 EPCs and P5 EPCs at 0, 8, and 12 h. Scale bars indicate 500 μm. (**D**) Fluorescence images showing the Dil-Ac-LDL uptake of EPC at P3 and P5 with scale bars indicating 100 μm (10×). (**E**-**F**) Quantitative analysis of unhealed area (E, *n* = 3) and Dil-Ac-LDL uptake (F, *n* = 5) of P3 and P5 EPCs. Student’s *t*-test. *p < 0.05, **p < 0.01, ***p < 0.001, ****p < 0.0001. (**G**) Time-lapse microscopy analysis of average speed. *n* = 50. Student’s *t*-test. *p < 0.05, **p < 0.01, ***p < 0.001, ****p < 0.0001. (H) Time-lapse microscopy analysis of total migration distance. *n* = 50. Student’s *t*-test. *p < 0.05, **p < 0.01, ***p < 0.001, ****p < 0.0001

We evaluated the morphological features and surface marker expression profiles of EPCs across P1 to P5 generations. We observed that P5 cells lost the characteristic morphology of EPCs and adopted a network-like growth pattern, as depicted in Fig. 2F. The percentage of VEGFR2 in P5 EPCs was drastically reduced to 5.38%, as shown in Fig. 3A. Subsequently, we investigated the functional properties of EPCs in different passages, focusing on tube formation, cell migration, and Ac-LDL uptake of EPCs. P3 EPCs demonstrated the highest forming extensive and well-connected tubular networks compared to other passages (Fig. 5A-B and Supplemental Fig. 3A-B). P5 EPCs failed to form complete tubes, in contrast to the P3 EPCs (Fig. 5A-B). P3 EPCs displayed the highest migratory ability, P2 and P4 EPCs showed moderate migration, and P1 EPCs exhibited limited migration (Fig. 5C and E, and Supplemental Fig. 3C-D). No migration was detected in P5 EPCs even after 12 h (Fig. 5C and E). The Ac-LDL uptake capacity of P5 cells showed significant functional decline compared to P3 EPCs (Fig. 5D and F). Time-lapse microscopy analysis revealed robust migratory capacity of P3 EPCs, while the P5 EPCs showed significantly decreased migration (Fig. 5G-H and Supplemental Fig. 3E). Quantitative tracking analysis showed P3 EPCs migrated at an average speed of 5.01 ± 5.2 μm/hr (Fig. 5G), with a mean total migration distance of 201.1 ± 32.2 μm over the observation period (Fig. 5H). These results demonstrate that P3 EPCs exhibited optimal biological functionality and proliferative capacity, while P5 EPCs exhibited a substantial decline in both purity and functional capacity compared to other generations.

### EPCs showed good wound-repair function in mice

**Fig. 6:**
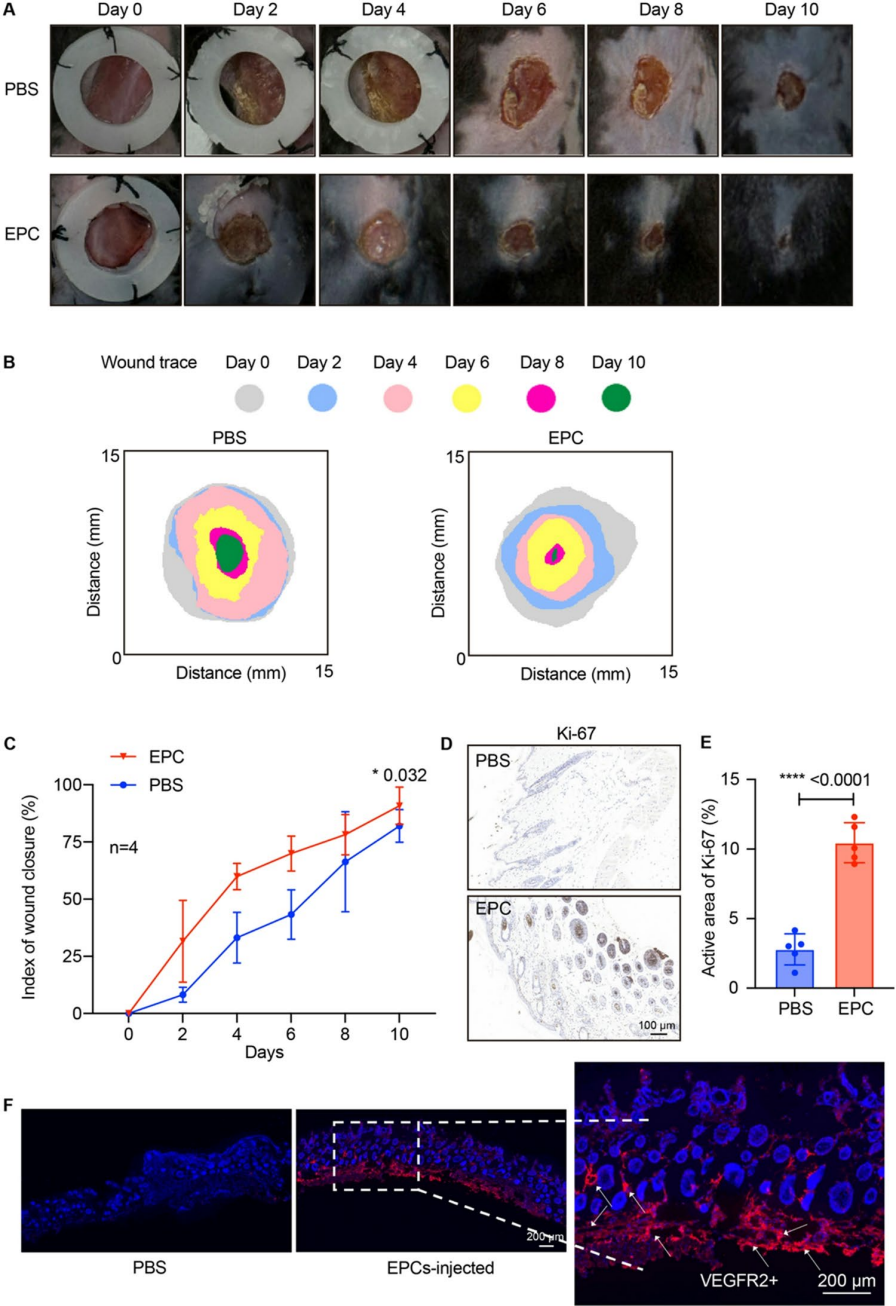
Wound healing assay demonstrating the effect of EPCs on wound closure. (**A**) Representative images of wound areas treated with PBS (control) and EPCs over a 10-day period (Day 0 to Day 10). (**B**-**C**) Quantitative analysis of wound closure, showing the distance of wound traces and the index of wound closure (%) over time. *n* = 3. Student’s *t*-test. *p < 0.05, **p < 0.01, ***p < 0.001, ****p < 0.0001. (**D**-**E**) Representative images (**D**) of immunohistochemical Ki-67 staining and quantification of Ki-67 (**E**) in wound tissues. Scale bars indicate 100 μm. *n* = 5. Student’s *t*-test. *p < 0.05, **p < 0.01, ***p < 0.001, ****p < 0.0001. (**F**) Immunofluorescence staining of human VEGFR2 (red) in wound tissues. Scale bars: 200 μm. The data illustrate the enhanced wound healing potential of EPCs compared to the PBS control

To assess the in vivo repair function of EPCs, we developed a skin wound mouse model. Circular excisional wounds were created on the dorsal skin of mice. Mice received a single tail vein injection of either PBS or EPCs (1.0 × 10^5^). Wound size was monitored every other day from day 0 to day 10 (Fig. 6A). Analysis of wound area demonstrated that EPCs-treated mice showed accelerated wound healing compared to controls (Fig. 6B-C). Ki-67 staining showed 4-fold higher proliferation in EPCs-treated wounds than the PBS control group (Fig. 6D-E). EPCs treatment significantly attenuated wound inflammation, reducing Ly6G^+^ (Neutrophil) by 48.94%, CD68^+^ (macrophage) by 53.4%, TNF-α by 52.17%, and IL-1β by 38.29% versus PBS controls (Supplemental Fig. 4A-D) (*p* < 0.05). Also, the injected human VEGFR2^+^ EPCs were detected in the wound, which suggested the persistent physical integration of human EPCs in mouse tissues (Fig. 6F). These findings suggested that EPCs significantly enhanced wound repair in vivo.

## Discussion

EPCs possess significant therapeutic potential and have been extensively utilized in numerous clinical trials for a wide range of diseases and disorders, including vascular diseases, wound healing, bone marrow repair, and graft-versus-host disease [11, 24, 25]. However, the number of circulating EPCs in healthy individuals is relatively low, and numerous factors can further influence this number [26]. The primary limitations to the clinical application of EPCs include insufficient cell quantity and purity, as well as the reliance on FBS for cell culture. Firstly, various EPC-enriched cell fractions, primarily derived from adult peripheral blood or bone marrow, have been utilized. However, the majority of EPCs obtained from these sources are heterogeneous populations [27, 28]. Secondly, the high cell doses required, coupled with the low frequency of EPCs, pose significant challenges for translating EPCs-based therapies to clinical settings. Moreover, in vitro expansion of cells is often required, and current protocols predominantly rely on the use of FBS [9]. The composition of FBS is variable and poorly defined, significantly influencing cell behavior and posing a critical challenge for consistent cell culture [7]. Therefore, the use of animal-origin components in cell culture represents a major barrier to the clinical translation of EPCs-based therapies. To realize the clinical application of EPCs, the three problems above must be addressed.

Our study successfully addressed these challenges, as the EPCs cultured in our animal-free system demonstrated high quality. Multiple parameters were analyzed to evaluate EPCs behavior, including cell morphology, growth characteristics, expansion potential, and the expression of typical EPCs phenotypic markers (positive) and unrelated markers (negative). The purity of EPCs cultured in our FBS-free system exceeded 95%, as determined by the expression of positive markers CD133, CD34, and VEGFR2, while CD31 and CD45 were consistently negative [26]. This high purity was maintained through the fourth generation (Fig. 3A-B). Notably, 1.04 × 10^8^ EPCs were obtained from just 10 g of placental tissue, demonstrating the high efficiency of our expansion system (Fig. 4B). Compared to bone marrow-derived EPCs [10], our placental EPCs demonstrated higher yield and enhanced stability (Purity > 95% maintained from P2 to P4). This suggests tissue-specific advantages for clinical scaling. The EPCs also exhibited robust capabilities, including Ac-LDL uptake, cell migration, and the formation of tubular structures (Fig. 4C-G). Furthermore, a single injection of EPCs significantly enhanced skin wound repair in our mouse model (Fig. 6A-C). Our internal control strategy using passage-matched cells provides clinically relevant quality thresholds, as recommended for cell therapy products. The obviously decreased function and purity of P5 EPCs clearly establishes the passage limit for therapeutic application. The abrupt loss of VEGFR2 expression at P5 (Fig. 3A) suggests a transient progenitor state of EPCs and that early-passage EPCs may have superior therapeutic efficacy. Our study not only establishes an animal-free culture system but also provides critical insights into the functional identity of placental-derived EPCs. Human VEGFR2^+^ EPCs detected in the wound confirmed the persistent physical integration of human EPCs in mouse tissues (Fig. 6F).

The growing application of EPCs in clinical trials has created a demand for efficient and safe in vitro expansion strategies. Here, we established an animal-free system for culturing EPCs from human placental tissue, highlighting their potential for regenerative medicine applications. Future research should focus on elucidating the molecular mechanisms underlying the stem characteristics of EPCs and exploring their therapeutic utility in additional preclinical models.

## Conclusions

Our study developed a method for culturing EPCs from human placental tissue in an animal-free system. The EPCs exhibited robust self-renewal capacity and maintained their endothelial lineage commitment through multiple passages. Our findings suggested that EPCs represent a promising cellular material for clinical applications, offering a potential solution to the limitations associated with traditional EPCs expansion methods. Future studies should further optimize this system and explore its applicability in a broader range of therapeutic contexts.

## Electronic supplementary material

Below is the link to the electronic supplementary material.

Supplementary Material 1

## Data Availability

All additional files are included in the manuscript, and data will be made available on reasonable request by contacting the corresponding author.

## Abbreviations

bFGF: Basic fibroblast growth factor
EPCs: Endothelial progenitor cells
IGF: Insulin like growth factor
i.v: Intravenous injection
MNCs: Mononuclear cells
SPF: Specific pathogen-free
VEGF: Vascular endothelial growth factor
VEGFR2: Vascular endothelial growth factor receptor 2
