# Supplementary material for: Human placenta-derived endothelial progenitor cells: an animal-free culture system for efficient expansion
Source: Biol Res. 2025 Jul 2;58:44. doi: 10.1186/s40659-025-00625-2 (PMC12219708; doi:10.1186/s40659-025-00625-2)
Supplement: Supplementary file 1 — Supplementary Material 1 [file 40659_2025_625_MOESM1_ESM.docx]

Supplementary Data for Shengnan Yuan *et al*.: ***Human Placenta-Derived Endothelial Progenitor Cells: An Animal-Free Culture System for Efficient Expansion*** (Including supplementary methods and 4 supplementary figures).

**Supplementary methods**

**Time-Lapse Microscopy for Random Cell Migration Analysis**

Random cell migration was performed using the time-lapse microscopy (AF-100, Live Cell Analyzer, INVIEW, China). Place the device in the cell culture incubator (37°C, 5% CO₂). EPCs were seeded in 6-well plates at a density of 5×10⁴ cells/well and allowed to adhere for 5 hours. Prior to imaging, the medium was replaced to minimize background fluorescence. Time-lapse images were acquired every 15 min for 10 hours using a 10× objective, with 9 random fields recorded per well. Cell trajectories were tracked and analyzed using the tracking software (ImageJ with TrackMate).

**Glucose Uptake Assay**

The glucose uptake assay was performed using a commercial 2-Deoxy-D-glucose (2-DG) assay kit (S0554, beyotime, China) according to the manufacturer's protocol. Briefly, seed EPCs at 5×10⁴ cells/well in 12-well plates and culture overnight. Cells were serum-starved for 2 hours in glucose-free medium, then incubated with 2-DG (100 μM) for 20 min at 37°C. After washing, cells were lysed and the amount of accumulated 2-DG6P was measured spectrophotometrically at 412 nm.

**Lactate Production Assay**

The lactate concentration in cell culture supernatants was measured using a commercial Lactate Assay Kit (S0208, beyotime, China) following the manufacturer's instructions. Briefly, cells were cultured in phenol red-free medium for 4 hours, and the supernatant was collected. After deproteinization, samples were mixed with the reaction mix and incubated for 30 min at 37°C. Absorbance was measured at 570 nm using a microplate reader. Lactate concentrations were calculated using a standard curve and normalized to total cellular protein.

**Supplementary figures**

**
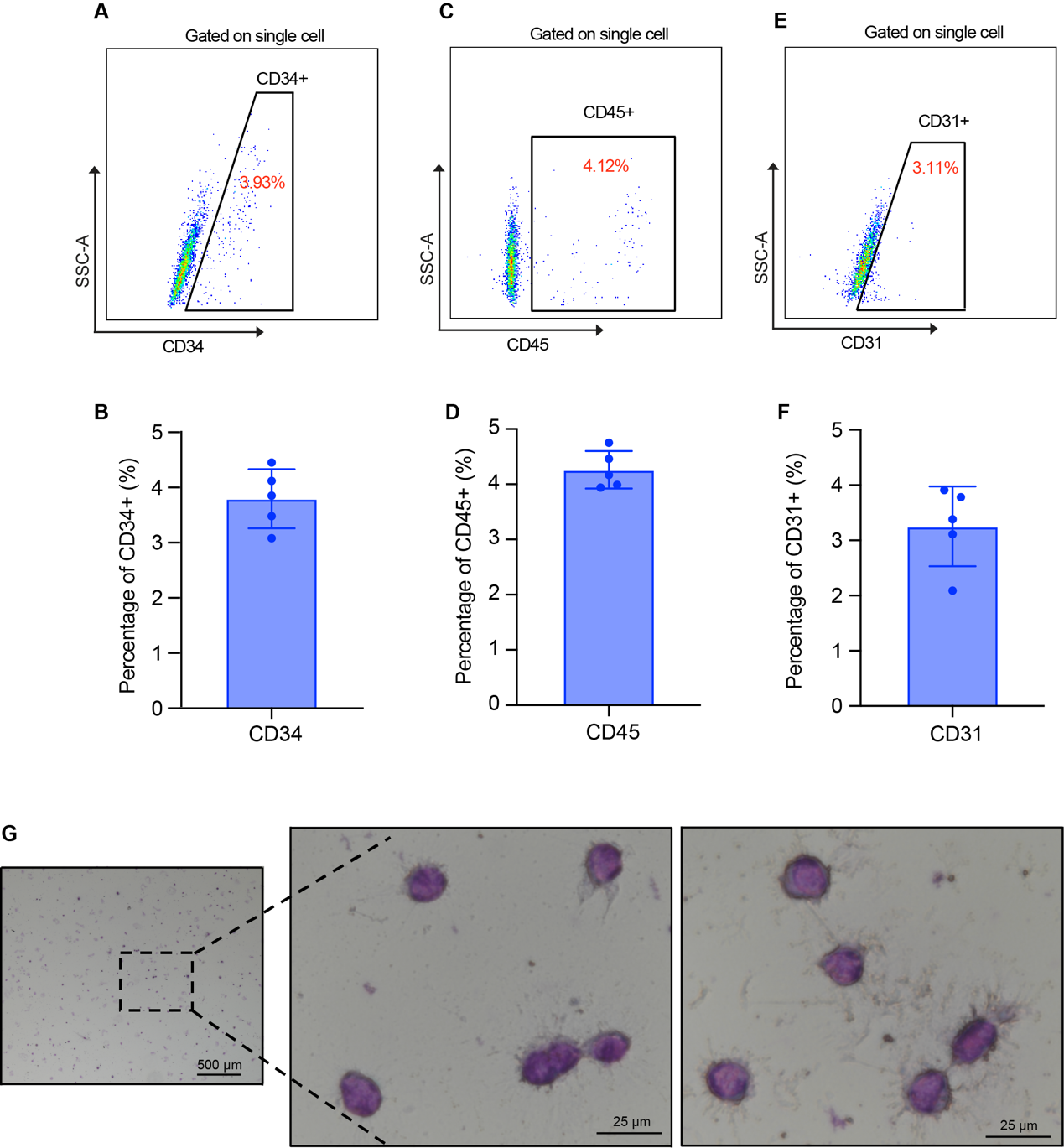
**

**Supplementary figure 1. The purity verification of MNCs from different placentas**

(A) Representative flow image of CD34. (B) Percentage of CD34 positive cells in MNCs from different placentas. n=5. (C) Representative flow image of CD45. (D) Percentage of CD45 positive cells in MNCs from different placentas. n=5. (E) Representative flow image of CD31. (F) Percentage of CD31 positive cells in MNCs from different placentas. n=5. (G) Representative Wright-Giemsa staining of isolated MNCs. n=5. Scale bars indicate 25 μm.


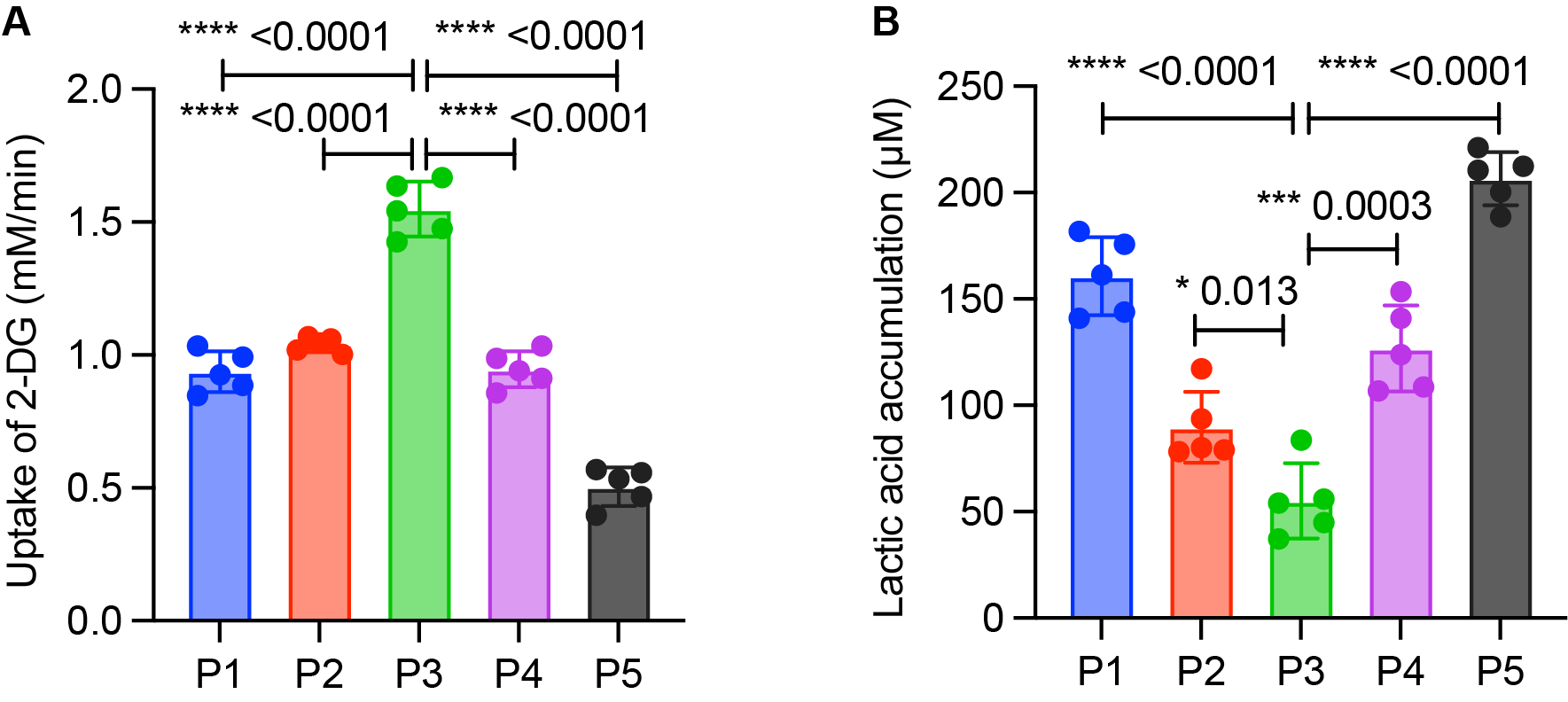


**Supplementary figure 2. Metabolic viability was different between P1-P5 EPCs**

(A) Glucose consumption rates of P1-P5 EPCs. (B) Lactate production of P1-P5 EPCs. n=5. Student’s *t*-test. **p* < 0.05, ***p* < 0.01, ****p* < 0.001, *****p* < 0.0001.


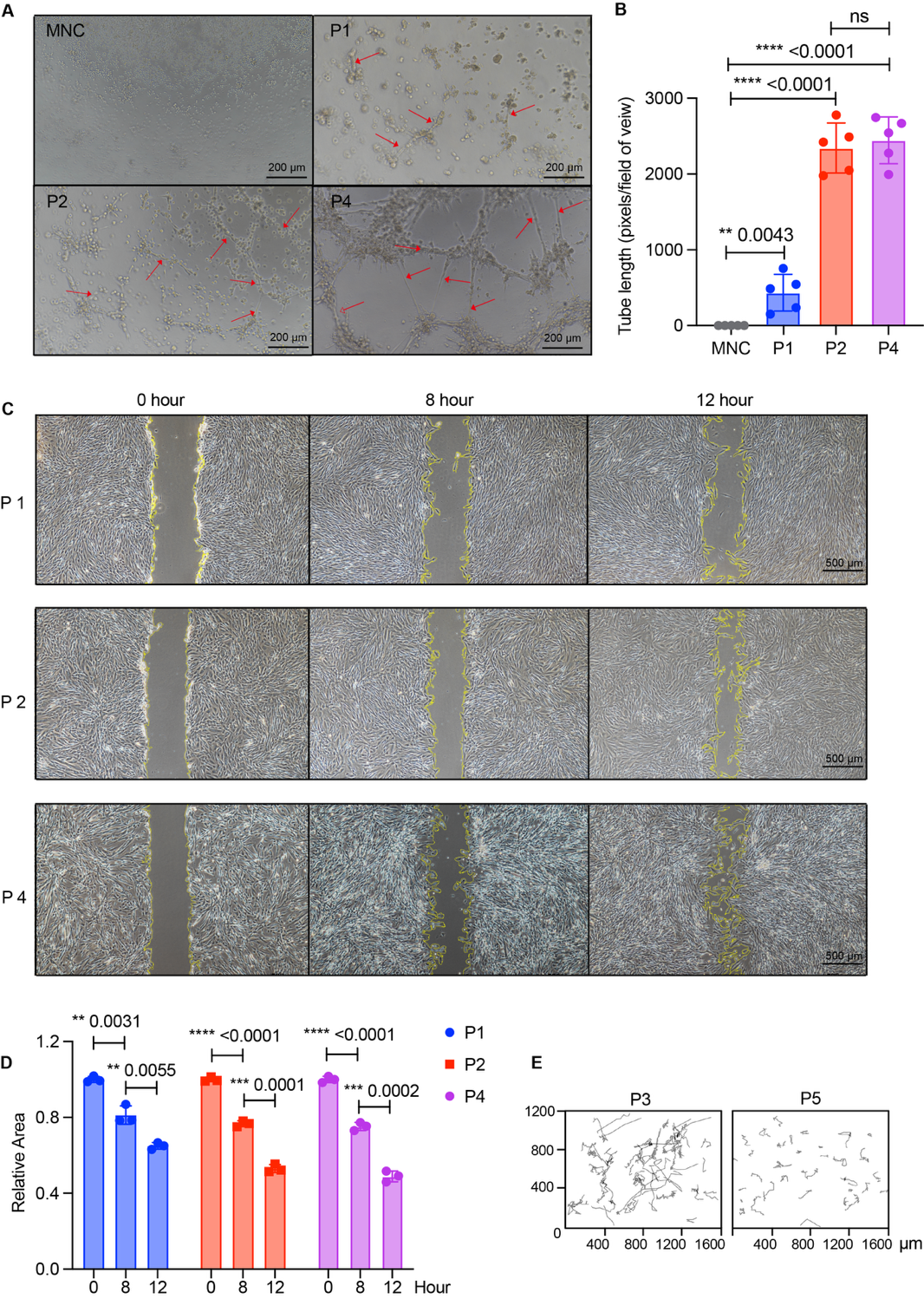


**Supplementary figure 3. The function of EPCs varied in different generations**

(A-B) Tube formation assay: Representative images (A) of tube formation (pixels of tubes per field of view) and quantification of the tube length (B) of MNCs, P1, P2, and P4 EPCs, with scale bars indicating 200 μm. n=5. Student’s *t*-test. **p* < 0.05, ***p* < 0.01, ****p* < 0.001, *****p* < 0.0001. (C) Cell migration assay: Representative images of P1, P2, and P4 EPCs at 0, 8, and 12 hours, with scale bars indicating 500 μm. (D) Quantitative analysis of unhealed area. n=3. Student’s *t*-test. **p* < 0.05, ***p* < 0.01, ****p* < 0.001, *****p* < 0.0001. (E) Diagram of the cell migration trajectory.

**
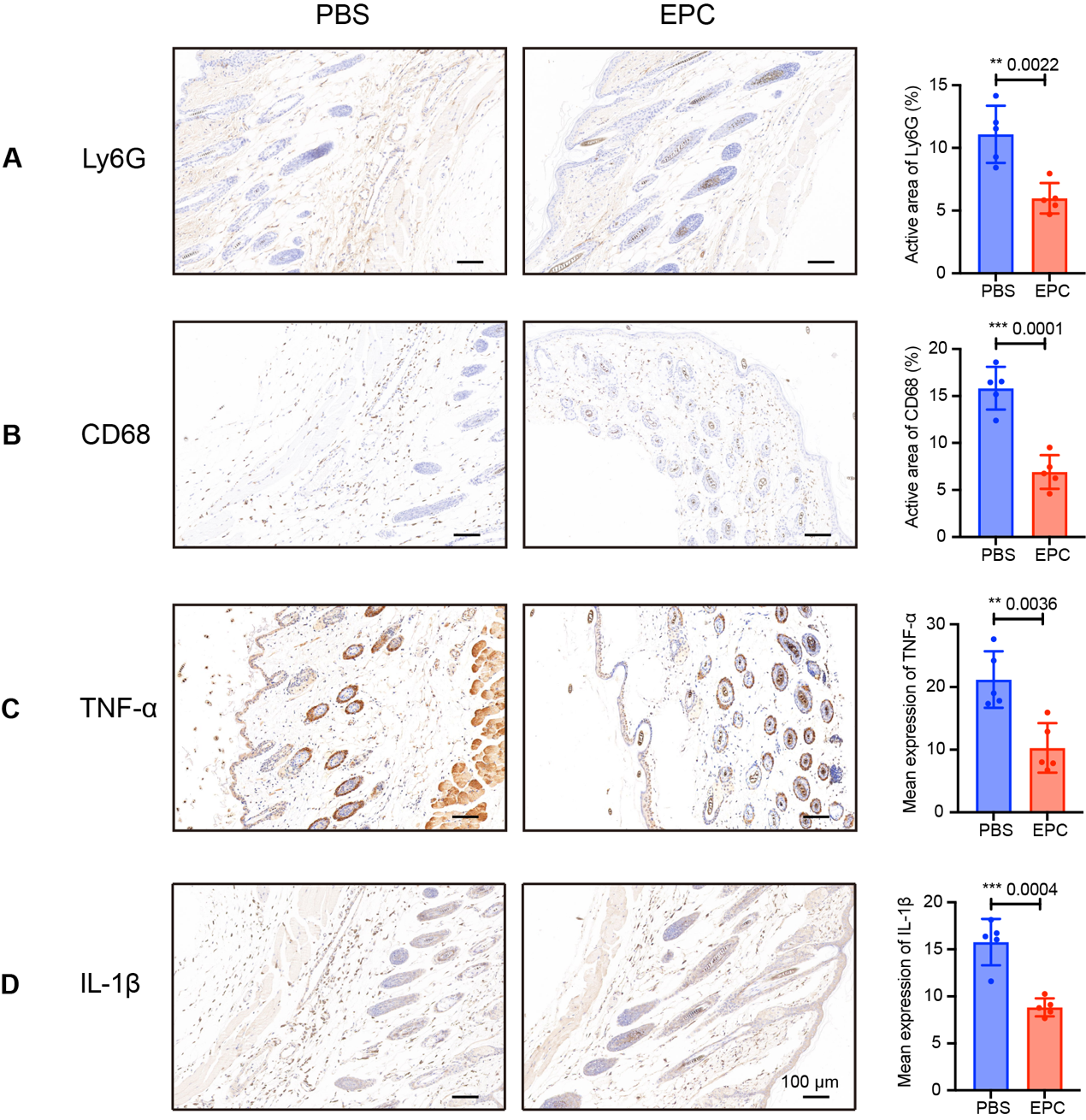
**

**Supplementary figure 4. EPCs treatment significantly attenuated wound inflammation**

(A) Representative images of immunohistochemical Ly6G^+^ staining (**Neutrophil)** and the quantification of Ly6G^+^ in wound tissues. (B) Representative images of immunohistochemical CD68^+^ staining **(macrophage)** and the quantification of CD68^+^ in wound tissues. (C) Representative images of immunohistochemical TNF-α^+^ staining and the quantification of TNF-α^+^ in wound tissues. (D) Representative images of immunohistochemical IL-1β^+^ staining and the quantification of IL-1β^+^ in wound tissues. Scale bar indicates 100 μm. n=5. Student’s *t*-test. **p* < 0.05, ***p* < 0.01, ****p* < 0.001, *****p* < 0.0001.
